# The effects of stroboscopic visual training on human cognitive function and motor performance: a systematic review

**DOI:** 10.3389/fphys.2025.1708783

**Published:** 2025-11-27

**Authors:** Yinghui Ren, Jingfu Zhang, Xiangpeng Sun, Fengxue Qi, Feng Guo

**Affiliations:** 1 College of Exercise and Health, Shenyang Sport University, Shenyang, China; 2 Sports, Exercise and Brain Sciences Laboratory, Sports Coaching College, Beijing Sport University, Beijing, China

**Keywords:** stroboscopic visual training, cognitive function, motor performance, neuroplasticity, sports training

## Abstract

This literature review examines systematically the effects of Stroboscopic Visual Training (SVT) on cognitive function and motor performance in humans with an integrated focus on the underlying neural mechanism. Systematic searches were conducted in major databases, including PubMed and Web of Science, and stringent screening procedures were applied. As a result, 35 high-quality experimental studies were identified and selected for in-depth analysis. The findings indicate that SVT can effectively and significantly enhance the speed of visual information processing and optimize core cognitive functions such as the allocation of attention. In terms of athletic performance, multiple indicators show improvements with enhanced precision in hand-eye coordination and increased motor reaction capabilities. SVT demonstrates consistent and positive effects regardless of athletes’ skill levels. In-depth investigation into its mechanisms reveals that the effects are associated with intermittent visual occlusion, which can induce significant changes in neural plasticity, thereby influencing both cognitive and motor performance. Overall, SVT holds substantial potential as an effective intervention for enhancing cognitive and motor performance. However, current research is limited by insufficient diversity and representativeness in sample selection, a lack of standardized training protocols, and an inadequate exploration of the underlying neural mechanisms. Future studies should aim to optimize experimental design, expand research contexts, and utilize advanced neuroimaging techniques to further elucidate the mechanisms through which SVT exerts its effects. Such efforts will help to unlock the full application value of SVT in fields such as sports training and rehabilitation, providing a more robust scientific foundation for its widespread implementation.

## 1 Introduction

Vision plays a pivotal role in athletic performance (Bao et al., 2017; Lochhead et al., 2024b). Fine motor actions—such as executing a volleyball spike (Jafarzadehpur et al., 2007; Zhu et al., 2024), hitting a baseball (Higuchi et al., 2018; Klemish et al., 2018), or performing a basketball jump shot (Oudejans et al., 2005; Piras, 2024)—are heavily dependent on accurate visual input (Buscemi et al., 2024; Appelbaum and Erickson, 2018). The link between visual capabilities and motor performance is widely regarded as a critical factor in determining athletic success (Erickson, 2022; Laby and Appelbaum, 2021). In recent years, an increasing body of research has focused on enhancing athletic performance through visual-based interventions (Krigolson et al., 2015; Hadlow et al., 2018; Hülsdünker et al., 2018). Among these, SVT has emerged as a promising and increasingly studied technique within the domain of sports training (Carrolla et al., 2021; Jothi et al., 2025; Smith et al., 2024).

**FIGURE 1:**
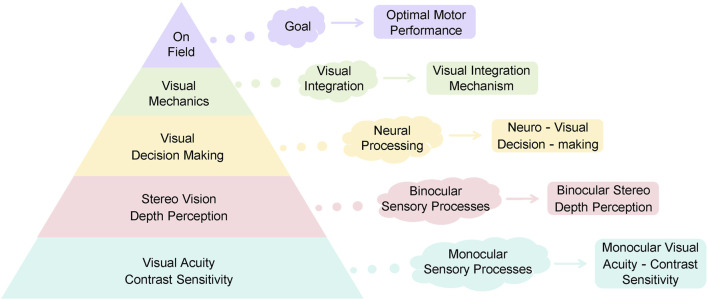
The Sports Vision Pyramid: A hierarchical model for athletic performance. The model proposes that on-field visual performance (apex) is built upon the neural integration of visual information (mid-layer), which itself processes inputs from fundamental visual sensory and mechanical skills (base layer).

SVT involves the use of stroboscopic eyewear during athletic activities (Das et al., 2023; Lochhead et al., 2024a). By alternating between transparent and opaque phases at adjustable frequencies, these glasses regulate the amount and continuity of visual information available to the athlete, thereby engaging neural circuits involved in visual and cognitive processing (Koppelaar et al., 2019; Wilkins and Appelbaum, 2020; Wilkins and Gray, 2015). This controlled visual disruption compels athletes to make decisions and respond under conditions of intermittent visual input (Braly and DeLucia, 2020; Erickson, 2021; Luo et al., 2025), which is theorized to enhance visual processing efficiency, improve motor performance (Vasile and Stănescu, 2023; 2024), and potentially reduce injury risk (Gokeler et al., 2018; Uzlaşır et al., 2021). According to the “Sports Vision Pyramid” model proposed by Kirschen and Laby (2011) (Figure 1), Attaining “optimal motor performance,” as situated at the apex of the Sports Vision Pyramid, depends on the integrity and efficiency of underlying neuro-visual processing functions at the foundational levels. Although SVT interventions may vary in their implementation, they consistently aim to enhance sport-specific skills and optimize athletic performance by improving visual-perceptual processing (Elliott and Bennett, 2021; Jothi et al., 2025).

Despite the demonstrated efficacy of stroboscopic vision in sports training, numerous questions remain unresolved. Indeed, the efficacy of SVT is modulated by multiple variables, including stroboscopic frequency (flashes per unit time), duty cycle (ratio of visual signal duration to cycle time), training duration, task specificity, and motor competence (Stănescu, 2021). However, current literature lacks standardized protocols for implementing and calibrating these variables across training paradigms.

This literature review systematically evaluates the effects of SVT on cognitive functions and motor performance, explores its underlying mechanisms, and analyzes key training parameters—including session frequency, duration, and stroboscopic rate—across various sports contexts and experimental conditions. It is anticipated that the findings from this review will facilitate the effective integration of SVT into athletic training and provide both theoretical insights and practical guidance for future research and performance enhancement.

## 2 Research methods

### 2.1 Literature search strategy

The design and reporting of this systematic review followed the Preferred Reporting Items for Systematic Reviews and Meta-Analyses (PRISMA) guidelines. To ensure comprehensiveness, a systematic search was conducted across multiple authoritative databases, including PubMed, Web of Science, Scopus, Google Scholar, and Ebsco. The search period ranged from the inception of each database to August 2024.

The search strategy was constructed based on the PICO framework and utilized Boolean operators to combine key terms. For example, the search strategy used in PubMed was as follows: (stroboscop*[Title/Abstract] OR “stroboscopic visual training” [Title/Abstract] OR “stroboscopic training” [Title/Abstract] OR “stroboscopic vision” [Title/Abstract] OR “stroboscopic glasses” [Title/Abstract] OR “shutter glasses” [Title/Abstract] OR “visual occlusion” [Title/Abstract]) AND (“Athletic Performance” [MeSH] OR “Sports” [MeSH] OR “Motor Skills” [MeSH] OR “Cognition” [MeSH] OR “Psychomotor Performance” [MeSH] OR “sports performance” [Title/Abstract] OR “motor performance” [Title/Abstract] OR “cognitive function” [Title/Abstract] OR “sports vision training” [Title/Abstract]).

### 2.2 Inclusion and exclusion criteria for literature

#### 2.2.1 Inclusion criteria

1. Population: Healthy human participants, with no restrictions on age, sex, or athletic proficiency.
2. Intervention: The primary intervention involved SVT. No restrictions were placed on intervention duration, session length, or stroboscopic frequency to allow for a comprehensive review of all existing protocols.
3. Study Design: Experimental studies, including randomized controlled trials and non-randomized trials.
4. Outcomes: Studies must report at least one objective measure of cognitive function or motor performance.

#### 2.2.2 Exclusion criteria

1. Intervention: Studies that did not implement SVT as an intervention.
2. Animal studies; studies involving clinical populations with known cognitive or motor impairments.
3. Publication Type: Review articles, conference abstracts, commentaries, or duplicate publications.

### 2.3 Literature screening process and quality assessment

#### 2.3.1 Study selection process

**FIGURE 2:**
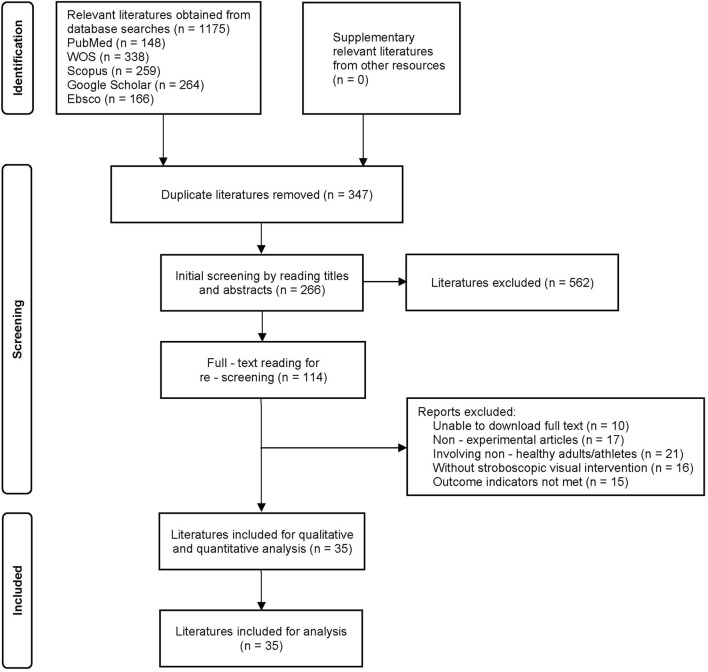
The search, screening and selection processes for suitable studies–based on PRISMA.

The literature screening process strictly adhered to the PRISMA guidelines, as detailed in Figure 2. The specific steps were as follows: Initially, 1,175 records were identified through database searching. After importing into the EndNote reference management software, 347 duplicate records were removed through both automated and manual processes. Subsequently, the titles and abstracts of the remaining 828 records were independently screened by two reviewers, leading to the exclusion of 562 records that did not meet the inclusion criteria. For the 266 records that passed the initial screening, the full texts were retrieved and reviewed, and then re-screened against the pre-defined inclusion and exclusion criteria. During this full-text screening phase, 81 records were excluded for reasons including inability to retrieve the full text, non-experimental articles, ineligible study populations, interventions not involving SVT, or outcome indicators not meeting the criteria. Ultimately, 35 studies were included for qualitative analysis (Zwierko M. et al., 2023).

#### 2.3.2 Risk of bias assessment

To assess the methodological quality of the included studies, the Cochrane risk-of-bias tool was employed. This assessment was conducted independently by two reviewers. Any disagreements were resolved through discussion or, if necessary, by consulting a third senior researcher.

**FIGURE 3:**
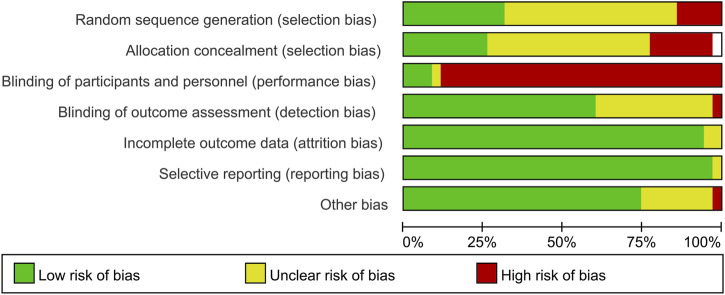
A bar chart summarizing the risk of bias for included studies across seven domains. Each bar represents the percentage of studies judged as low, unclear, or high risk for a specific domain. The majority of studies show low risk in “Random sequence generation”, “Allocation concealment”, “Incomplete outcome data”, and “Other bias”. In contrast, most studies have high risk in “Blinding of participants and personnel”. Judgments for “Blinding of outcome assessment” and “Selective reporting” are predominantly unclear.

The methodological quality of the included studies was evaluated using the Cochrane Risk of Bias tool, with a summary of the results presented in Figure 3. Overall, the majority of studies demonstrated a low risk of bias in domains related to selection bias, such as random sequence generation and allocation concealment. However, a significant proportion of studies were judged as having a high risk of performance bias (blinding of participants and personnel), which is largely inherent to the nature of the SVT intervention. The risks of detection bias (blinding of outcome assessment) and reporting bias (selective reporting) were most frequently rated as unclear for a majority of the studies. Risks associated with attrition bias and other biases were generally low. This assessment indicates that the core quality of the evidence synthesized in this systematic review is acceptable, despite inherent limitations related to the intervention’s characteristics.

## 3 Results

### 3.1 Data extraction and synthesis methods

#### 3.1.1 Data extraction

A standardized data extraction form was developed. Two reviewers independently extracted key information from each included study, encompassing: (a) study characteristics (author, year, design); (b) participant profiles (sport, skill level); (c) SVT intervention parameters (frequency, duration, stroboscopic rate, duty cycle); (d) outcome measures related to cognitive function and motor performance; and (e) main findings.

#### 3.1.2 Data categorization and synthesis

**FIGURE 4:**
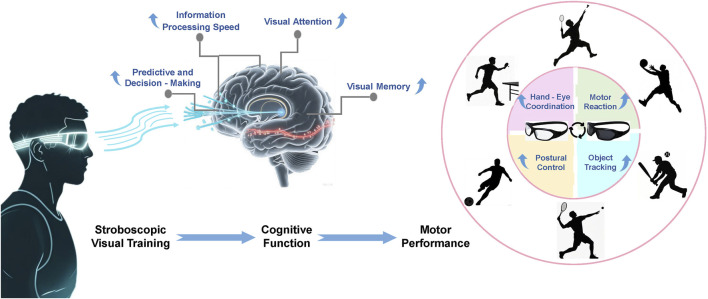
The mediating role of cognition between Stroboscopic Visual Training (SVT) and motor performance. The model illustrates how SVT may primarily target cognitive functions, which in turn serve as the mechanism for improving specific motor skills.

The extracted findings were categorized and synthesized by key outcome domains (e.g., information processing speed, visual attention, hand-eye coordination, motor reaction time). Within each domain, studies were systematically compared and contrasted to identify consistent patterns, divergent results, and the strength of the evidence. This narrative summary approach, presented in the following sections and summarized in Tables 1, 2, forms the basis for the conclusions drawn in this review. The conceptual model illustrating the mediating role of cognition (Figure 4) was developed to visually synthesize the central mechanism derived from this analytical process.

**TABLE 1:** Research protocols and main findings of the effects of stroboscopic visual training on cognitive function.

Article	Sport	Sport population/ level	Research objective	Training frequency	Stroboscopic frequency (Hz)	Visual perception and cognitive measurement	Post - effect assessment	Main findings
Elhachemi and Issam, (2024)	Football	Amateur level	To investigate the impact of SVT on basic skills (e.g., ball control and reaction speed) in football players	1 week 2 sessions/week, training duration unspecified	Not specified	1. Professional reaction test 2. Nelson sports reaction test	–	SVT significantly improved players’ reaction speed
Zwierko M, et al. (2023)	Volleyball	Professional adolescent players	To explore the impact of *in-situ* stroboscopic training on visual, visuomotor, and reactive agility in adolescent volleyball players	6 weeks 3 sessions/week, training duration unspecified.	Week 1: 15 Hz, 50%* Week 2: 13 Hz, 50%* Week 3: 11 Hz, 50%* Week 4: 10 Hz, 50%* Week 5: 9 Hz, 60%* Week 6: 9 Hz, 70%*	1. Vienna Test system S1 for simple reaction speed 2. Determination test S1 for complex reaction speed 3. Flash fusion frequency test for sensory sensitivity threshold 4. Free visual search task for scanning reaction	4 weeks	1. SVT yielded greater improvements in visual and visuomotor functions than conventional training 2. SVT significantly improved 3 of 5 measured visual/visuomotor functions, particularly in visuomotor (vs. Sensory) processing 3. SVT may enhance visual information processing efficiency, indirectly training eye movement control as evidenced by improved scanning dynamics
Hülsdünker T, et al. (2021a)	Badminton	Elite youth players	To examine short-term and long-term effects of SVT on visuomotor reaction speed in elite young badminton players	10 weeks ≥1 session/week 25–30 min total (10–15 min/session)	Week 1: 15 Hz, 50%* Week 2: 13 Hz, 50%* Week 3: 11 Hz, 50%* Week 4: 10 Hz, 50%* Week 5: 10 Hz, 60%* Week 6: 9 Hz, 60%* Weeks 7–8: 9 Hz, 70%* Weeks 9–10: 8 Hz, 70%*	Radial movement test	6 weeks	1. SVT significantly reduced visuomotor reaction time, with immediate post-training effects and 6-week retention 2. SVT improved reaction speed primarily by shortening pre-movement time (i.e., from stimulus presentation to muscle activation), rather than movement time (i.e., from muscle activation to movement execution)
Ballester R, et al. (2017)	–	College students	To examine the impact of stroboscopic visual conditions on coincident anticipation performance over time and attention allocation during task execution	Single-session training	4 Hz	Psychomotor vigilance task (PVT)	–	1. Stroboscopic visual conditions facilitated better attention maintenance 2. Short-term SVT exhibited positive effects, but prolonged attention load induced cognitive fatigue
Mitroff SR, et al. (2013)	Ice hockey	Elite level	To determine whether SVT directly enhances skill performance in professional ice hockey players	16 consecutive days, ≥10 min/day	1–6 Hz	Position - specific skill test	–	SVT improved visual ability, attention, and reaction time
Appelbaum LG, et al. (2011)	–	Soccer (athletes), frisbee (amateur enthusiasts), general individuals	To investigate whether SVT improves visual - cognitive ability and achieves generalized learning, enabling participants to apply new skills/strategies flexibly in untrained tasks/situations	Single - session (general individuals) 2 weeks, 2 sessions/week (frisbee) 9–10 sessions within 12 days (soccer)	1–6 Hz	1. Motion continuity threshold 2. Useful field of view - dual - target task 3. Multiple - target tracking	–	1. SVT significantly improved central visual field motion sensitivity and short - term attention 2. No significant effects on peripheral visual field motion sensitivity, peripheral short - term attention, or sustained attention (multiple - target tracking task)
Beavan A, et al. (2021)	Soccer	Amateur level	To study the impact of visual feedback restriction on soccer - specific skills, particularly the coupling of soccer - specific perceptual information and motor actions	2 days, training duration unspecified	Full - field 1 Hz	Footbonaut system for specific skill assessment	2 days	Visual restriction forced athletes to reallocate attention resources, affecting information - processing speed and accuracy
Müller S, et al. (2024)	Soccer	Professional athletes	To investigate whether skilled athletes can maintain skill performance during periodic visual information interruption	Single-session training	Full-field 6 Hz 2.25 Hz 1 Hz	Self-reported attention focus during shooting tasks	–	When visual information was limited, athletes dynamically adjusted attention focus between external environmental and internal technical cues
Appelbaum LG, et al. (2012)	–	College students, school-team soccer/basketball players	To investigate the impact of SVT on visual memory (particularly information encoding in short-term memory)	Experiment 1: 2–7 sessions Experiment 2: Single-session	1–6 Hz	1. Memory retention test 2. Motion coherence task	1 day	1. SVT enhanced visual memory information retention 2. Experiment 1 showed significant short-term memory improvement without altering sensory memory duration 3. Experiment 2 demonstrated SVT-induced visual memory improvement lasting ≥24 h 4. SVT effects generalized beyond specific training tasks
Palmer T, et al. (2022)	Soccer	Club youth players	To investigate the effect of a 4-week SVT program on dribbling performance in young soccer players	4 weeks 1 session/week 20 min/session	Week 1: 4 Hz Week 2: 3 Hz Week 3: 2.25 Hz Week 4: 1.75 Hz	1. Central motion sensitivity 2. Short-term memory retention 3. Anticipation timing 4. Dynamic visual acuity	1 week	SVT acutely influenced players’ dribbling decision-making strategies, but this effect did not persist in the 1-week retention test
Fortes et al. (2025)	Soccer	Professional athletes	To examine the impact of repeated SVT on multiple-target tracking, anticipation, and decision-making skills in soccer players	8 weeks 3 sessions/week 20 min/session	4.76 Hz	1. Multiple-target tracking task 2. Game-based anticipation and decision-making assessment	–	1. The SVT group showed greater improvement in anticipation task accuracy than the control group 2. Both groups exhibited reduced cognitive load during anticipation tasks (measured by pupil diameter)
Top E. (2022)	Soccer	Professional athletes	To evaluate the impact of 3D object tracking and stroboscopic training on perceptual-cognitive performance and sport-specific decision-making in soccer players	8 weeks 2 days/week 40–55 min/session	1–6 Hz	1. Stroop test 2. Deary-liewald reaction time test 3. Stop signal test 4.3D object tracking task	–	1. Significant improvements in perceptual-cognitive test performance, sport-specific decision-making accuracy, and decision consistency were observed 2. Training effects transferred from the laboratory to real-game environments
Boiangin NM. (2019)	Tennis	Intermediate-level players	To investigate the impact of SVT on anticipatory response accuracy, reaction time, and self-efficacy in serve direction prediction among intermediate-level tennis players	6 consecutive days 1 session/day 30 min/session	Full-field 10 Hz 1 Hz	1. Watch serve videos and predict serve direction (left/right) as quickly as possible after racket-ball contact occlusion 2. Self-efficacy rating scale	1 week	1. The low-frequency (1 Hz) training group demonstrated significantly higher serve direction prediction accuracy than the other groups in the 1-week follow-up 2. Participants’ self-efficacy increased under all training conditions, with no significant differences between conditions
Li T, et al. (2024a)	Curling	Elite-level athletes	To examine the impact of SVT on key performance metrics in elite curlers and determine whether these effects can be utilized in long-term training to enhance performance	Single-session training	Full-field 2.5 Hz	Watch videos of curling stones moving in a specific pattern and determine the moment the stone’s front end touches the T-line	–	1. Stroboscopic visual conditions had negative impacts on cognitive functions, particularly time perception and judgment errors 2. Throwing speed control and accuracy performance significantly declined under stroboscopic conditions
Li T, et al. (2024b)	Curling	Elite-level athletes	To investigate the effects of a 4-week SVT combined with time-feedback training (TFT) on time-judgment ability and throwing performance (including speed control and accuracy) in elite curlers	4 weeks 3 sessions/week 40 min/session	Week 1: 6 Hz Week 2: 5 Hz Week 3: 4 Hz Week 4: 3 Hz	Watch videos of curling stones moving in a specific pattern and determine the moment the stone’s front end touches the T-line	–	Both SVT and TFT significantly improved time-judgment and throwing speed control in elite curlers. SVT showed greater effects on time-judgment and speed control, while TFT was more effective in improving throwing accuracy

*Represents the duty cycle, that is, the proportion of time per cycle during which the lens of the stroboscopic glasses is transparent; PVT, psychomotor vigilance task; TFT, time feedback training.

**TABLE 2:** Research protocols and main findings on the effects of stroboscopic visual training on motor performance.

Article	Sport	Sport population/level	Research objective	Training frequency	Stroboscopic frequency (Hz)	Visual perception and cognitive measurement	Post - effect assessment	Main findings
Oudejans RR, (2012)	Basketball	Elite-level female players	To investigate the effect of visual control on shooting performance in elite female basketball players	3 months 1–2 sessions/week 15–20 min/session	Not specified	1. Shooting percentage 2. Total shooting completion time	–	1. SVT significantly improved shooting percentage, with improvements emerging gradually during training 2. Players exhibited faster shooting action execution speeds
Liu S, et al. (2020)	Baseball	College professional athletes	To test whether dynamic visual training improves hitting performance in college baseball batters, including enhancing performance through vision, perception, and oculomotor ability training	10 weeks 1–2 sessions/week 30 min/session	1–6 Hz	Trackman Doppler radar or HitTrax system measurements: Exit velocity, launch angle, hitting distance, batting average, on-base percentage, slugging percentage, walk rate, strikeout rate	–	Dynamic visual training positively impacted batting practice performance, particularly in hitting angle and distance
Ellison P, et al. (2020)	–	College and national-level athletes	To determine whether SVT improves hand-eye coordination performance in athletic practice tasks	Single-session training	6 Hz	1. Visual search test 2. Hand-eye coordination test using sport vision Trainer™ 80-sensor board	10 min, 10 days	1. Single-session SVT significantly improved hand-eye coordination immediately, 10 min post-training, and in the 10-day retention test 2. SVT effects were task-specific, improving hand-eye coordination without transfer to visual search tasks
Edgerton L, (2018)	Softball	Beginners and professional athletes	To investigate the impact of SVT on ball-catching and hitting abilities in softball players, and to explore whether beginners exhibit greater improvement potential than professionals	4 weeks, 3 sessions/week, training duration unspecified.	6 Hz	1. Ground ball fielding performance 2. Subjective reports on visual and perceptual skills	–	1. SVT improved ground ball fielding ability in softball players 2. Novice players showed greater improvement margins than expert players 3. Players reported enhanced ball-catching confidence, self-esteem, and reduced effort/tension levels
Jendrusch G, et al. (2023)	Tennis	High-level and medium-low-level players	To analyze the use of stroboscopic glasses in kinesthetic perception training and their impact on hitting accuracy, as well as the effects of frequency/duty-cycle ratios on hand-eye coordination	Single-session training	Full-field 5.3 Hz 2.3 Hz 1 Hz	1. Target-area hitting accuracy 2. Subjective assessments of perceptual limitation	–	1. Reduced visual perception negatively affected target hitting accuracy in specific forecourt interception scenarios 2. High-level players demonstrated higher accuracy and stronger anti-interference ability under all stroboscopic conditions
Wilkins L, et al. (2018)	Soccer	Elite-level young goalkeepers	To examine the impact of SVT on visual and perceptual skills in three elite young soccer goalkeepers	7 weeks 2 sessions/week 45 min/session Pre-post test: 1 session (5 min training)	1–6 Hz	1. Tennis ball catching practice 2. Goalkeeper-specific soccer drills 3. Semi-structured interviews	4 weeks	1. The experimental group showed continuous improvement in visual reaction time during post-test and 4-week retention, while the control group did not 2. Participants reported improved reaction, judgment, and concentration through SVT.
Zwierko M, et al. (2024)	Volleyball	Professional athletes	To investigate the impact of 6-week SVT on visually guided reactive speed in target-oriented movements among young volleyball players	6 weeks 3 sessions/week 65 min/session	Week 1: 15 Hz, 50%* Week 2: 13 Hz, 50%* Week 3: 11 Hz, 50%* Week 4: 10 Hz, 50%* Week 5: 9 Hz, 60%* Week 6: 9 Hz, 70%*	1. Five-time shuttle run via fusion sport smart speed system 2. Visual search task using a portable eye-tracking system	–	1. SVT significantly enhanced visual reaction speed in target-oriented movements 2. Scanning dynamics may mediate the improvement in visual reaction speed
Fransen J, et al. (2017)	Soccer	High-level athletes	To examine the impact of stroboscopic glasses limiting visual feedback on dribbling performance in young soccer players	Single-session training	Full-field 4 Hz 1.33 Hz	Grand valley state university dribbling test	–	1. Visual feedback limitation increased dribbling test completion time for all ability levels 2. Fast dribblers showed the most pronounced performance decline under visual restriction 3. The initial stage of motor skill learning is highly dependent on available feedback
Strainchamps P, et al. (2023)	Table tennis	Top-level athletes	To determine whether stroboscopic glasses used during warm-up confer additional benefits for specific movement reaction speed in skilled table tennis players	Single 10-min session	Full-field 20 Hz, 50%*	High-speed ball target-hitting task	–	1. Stroboscopic glasses did not improve reaction time or ball-hitting time, with no immediate positive effects on visuomotor reaction performance during warm-up 2. No negative performance impacts were observed, indicating athletes can incorporate stroboscopic warm-ups without performance concerns
Zwierko T, et al. (2024)	Soccer	Second-level athletes	To investigate the short-term impact of stroboscopic stimulation during specific soccer warm-up stages on reactive agility (RA) in pre-planned and unpredictable tasks, and to test these effects in ball/ball-less and fatigued/non-fatigued conditions	Single-session training	15 Hz, 50%*	1. RA test via Fitlight® system 2. Change-of-direction speed (CODs) via Illinois agility test	–	1. Stroboscopic glasses used during ball-specific warm-up significantly improved RA in ball-handling and unpredictable stimulus tasks, independent of fatigue status 2. No significant differences were observed in ball-less RA tests, indicating task-specific effects of stroboscopic stimulation
McCreary ME, et al. (2024)	–	Healthy adults	To examine the impact of stroboscopic glasses on standing balance in spatio-temporal and frequency domains, and analyze the effects of different stroboscopic frequencies and training duration on static balance test indicators	14 consecutive days, 1 session/day, training duration unspecified	Not specified	1. Static balance tests: Single-leg stance test, tandem stance test 2. Dynamic balance tests: Balance beam walk test, balance board test	1 day; 3 days	1. The experimental group showed significant balance improvement post-training, while the control group showed no change 2. Twelve Hz was more effective than 10 Hz for balance improvement 3. Stroboscopic training effects persisted short-term but weakened over time, indicating the need for continuous training to maintain benefits
Kim KM, et al. (2017)	–	Healthy adults	To determine whether stroboscopic glasses induce sensory reweighting of visual input during single-leg balance testing	Single-session training	5 Hz	1. Single-leg stance test 2. Traditional center-of-pressure (COP) test	–	Stroboscopic vision induced sensory reweighting of visual input, particularly when somatosensory input was disrupted (e.g., on a foam pad)
Kim KM, et al. (2020)	–	Healthy adults	To evaluate whether stroboscopic vision (SV) can serve as an alternative to sway-referencing vision (SRV) for assessing postural stability	Single-session training	5 Hz	1. Clinical test of sensory integration and balance (CTSIB) 2. Time-to-boundary (TTB) test 3. Sensory organization test (SOT)	–	Postural instability induced by SV was comparable to that by SRV, demonstrating SV can serve as a convenient and cost-effective alternative for assessing sensory dependence and reorganization
Lee H, et al. (2022)	–	Healthy adults	To investigate the impact of stroboscopic glasses on visual dependence during postural control, particularly changes in visual dependence under different surface conditions and stroboscopic difficulty levels	Single-session training	Full-field 4 Hz 2.25 Hz	1. Single-leg balance test 2. Dynamic postural stability index (DPSI) test 3. Y-balance test (YBT)	–	Stroboscopic glasses altered visual dependence in static and dynamic postural control, especially when the somatosensory system was disrupted
Jang J (2019)	–	Healthy adults	To investigate the impact of different stroboscopic visual frequencies (full vision, high-frequency stroboscopic vision, low-frequency stroboscopic vision) on the relative contribution of visual information in balance control	Single-session training	1. Full vision) 2. High-frequency stroboscopic vision 3. Low-frequency stroboscopic vision	1. Somatosensory organization measure (SOM) 2. Sensory organization test (SOT) 3. Visual information utilization (VIS)	–	Reduced visual information (e.g., low-frequency stroboscopic vision) decreased reliance on visual input in balance control, prompting increased dependence on somatosensory and vestibular systems
Symeonidou and Ferris, (2022)	–	Healthy adults	To investigate whether intermittent visual occlusion can serve as a method to augment dynamic balance training efficacy	Single-session training	1 Hz	Balance beam walk test	2 weeks	1. SVT significantly improved dynamic balance ability, outperforming traditional non-visual occlusion training 2. SVT enhanced both short-term balance improvement and long-term retention 3. Balance enhancement occurred via multisensory integration (vision, vestibular, and somatosensory systems)
Chen Y-C, et al. (2025)	–	Healthy elderly	To examine the impact of stroboscopic vision (SV) on postural control in older adults, focusing on cortical-postural coupling and control changes during feedback/feedforward processes	Single-session training	1 Hz	Stable standing posture task	–	1. SVT prompted elderly individuals to rely on open-loop control for postural instability, indicating increased dependence on internal somatosensory information under limited visual feedback 2. SVT altered postural sway phase and cross-frequency coupling between scalp electroencephalogram and posture. Increased β-band PAC in frontal/sensorimotor regions correlated with postural control strategy shifts, suggesting neural mechanism adaptations
Tsai Y-Y, et al. (2022)	–	Healthy elderly	To investigate the impact of stroboscopic vision (SV) on postural sway dynamics and cerebral cortical responses during unstable standing in older adults	Single-session training	3 Hz	Dynamic sway platform postural control task	–	1. SV increased postural control difficulty on the sway platform for the elderly, inducing more regular postural sway, which indicated reduced reliance on visual input and increased dependence on non-visual perception 2. Decreased β-band power in the middle temporal lobe under SV suggested fewer error perceptions, while enhanced α-band supra-threshold connectivity indicated inhibition of visual spatial attention and dorsal visual stream functions
Holliday J. (2013)	Lacrosse	First-level athletes	To examine the impact of SVT on dynamic visual acuity scores	2 weeks 5 sessions/week 25 min/session	1–6 Hz	1. Dynamic visual acuity 2. Static visual acuity 3. Minimum perception time test 4. Ball-catching training	2 weeks	Visual short-term memory retention, perception ability, and ball-catching skills were significantly improved
Bennett SJ, et al. (2018)	–	Healthy adults	To compare the interference and elimination effects of different stroboscopic visual methods (stroboscopic glasses, PLATO visual occlusion, intermittent display) on visuomotor and form perception, and their impact on sensorimotor skill acquisition	Single-session training	5.6 Hz 3.2 Hz 1.8 Hz	1. Experiment 1: Multiple-object tracking (MOT) task 2. Experiment 2: Multiple-object avoidance (MOA) task	–	1. Stroboscopic visual conditions differentially affected MOT task performance: Stroboscopic glasses preserved visual motion and form, whereas PLATO visual occlusion and low-frequency intermittent display impaired participants’ ability to detect and relocate moving objects 2. Decreasing stroboscopic frequency increased attention allocation to the MOT task, but only the stroboscopic glasses group main tained performance through attentional modulation 3. Stroboscopic glasses training promoted acquisition of sensorimotor skills for maintaining and updating multiple moving objects under cognitive-motor conflict 4. The MOA task was too challenging for skill acquisition, but stroboscopic glasses balanced interference with visual motion/form to provide optimal learning challenges

*Represents the duty cycle, that is, the proportion of time per cycle during which the lens of the stroboscopic glasses is transparent; RA, reactive agility; CODs, Change-of-Direction Speed; COP, center of pressure; SV, stroboscopic vision; SRV, Sway-Referencing Vision; CTSIB, clinical test of sensory integration and balance; TTB, Time-to-Boundary; SOT, sensory organization test; DPSI, dynamic postural stability index; YBT, Y-Balance Test; SOM, somatosensory organization measure; VIS, visual information utilization; PAC, Phase-Amplitude Coupling; MOT, Multiple-Object Tracking; MOA, Multiple-Object Avoidance.

### 3.2 Summary of research protocols

#### 3.2.1 Overview of subjects included in the research

In the reviewed literature, the sample sizes of the included research subjects varied considerably. Most studies reported sample sizes of less than 40. Four studies had over 100 subjects, with two studies featuring 189 participants (Beavan et al., 2021; Fransen et al., 2017). Nine studies included fewer than 20 subjects, and two of them had less than 10 subjects, specifically 3 (Elhachemi and Issam, 2024) and 6 (Wilkins et al., 2018).

Regarding sports specializations, these studies predominantly covered football (9 studies), badminton (1 study), table tennis (1 study), volleyball (2 studies), tennis (2 studies), basketball (1 study), baseball (1 study), softball (1 study), rugby (1 study), ice hockey (1 study), and curling (2 studies).

Concerning the athletic levels of the subjects, the studies included amateurs, professionals, elites, top - level athletes, and university athletes, indicating the widespread application of this technology across different sports levels. Three studies that included subjects of varying sports levels pointed out that lower - level athletes have greater room for improvement (Edgerton, 2018; Ellison et al., 2020), while high - level athletes demonstrate more stable performance under stroboscopic intervention (stronger anti - interference ability) (Jendrusch et al., 2023).

#### 3.2.2 Summary of intervention measures

All studies provided detailed reports on specific intervention measures and collected outcome - measure data at least once before and after the intervention. In terms of the duration of the intervention, there was significant variation. The shortest was a single - session acute training program (n = 16). The remaining studies adopted longitudinal training interventions lasting from several days to weeks. The most common implementation frequency for these interventions was 2–3 times per week, with each session lasting 20–40 min. The duration of the intervention cycle mainly ranged from 4–10 weeks, and the longest training intervention lasted for months, basically covering the entire season of a certain sport.

Regarding the stroboscopic frequency, it mainly ranged from 1–6 Hz, with a duty cycle (the percentage of the on - time in a cycle) ranging from 50% to 70%. For subjects with high - precision requirements and high - level athletes (such as in badminton), the training cycle was longer; and the stroboscopic frequency range could be extended to 8–15 Hz. Most studies conducted tests immediately after the training ended. Thirteen studies also conducted follow-up evaluations at various time points: 10 min (1 study), 1 day (2 studies), 2 days (1 study), 3 days (1 study), 1 week (2 studies), 10 days (1 study), 2 weeks (2 studies), 4 weeks (2 studies), and 6 weeks (1 study) after the intervention.

In summary, the reviewed studies displayed substantial diversity in sample sizes, participant profiles, intervention durations, and stroboscopic parameters. This high degree of variability across protocols highlights a significant challenge in comparing results across studies and underscores the need for standardization in future SVT research.

#### 3.2.3 Summary of measurement results

The most common evaluation outcomes in these studies predominantly focused on behavioral performance related to specific sports stimuli and tasks. Specifically, nine studies employed sport - specific measurement methods, including completing sport - specific action tasks (such as penalty kicks, batting, and softball fielding) or perception - decision tasks based on computer - programmed scenarios (like predicting shooting actions from video clips). Additionally, three studies utilized non - sport - specific general stimulus tasks, with most involving computer - programmed cognitive tasks (such as multiple - object tracking). It is worth noting that some studies (n = 23) incorporated both sport - specific tasks and general stimuli as multi - dimensional outcome measurement indicators in their experimental designs. Moreover, five of the included studies utilized questionnaires to gather subjective evaluation data.

The results of this review support the notion that SVT can enhance skill levels and motor performance by improving human cognitive function (Figure 4). Specifically, 15 studies confirmed the association between the intervention effects of SVT and specific visual cognitive functions (such as visual information processing speed, visual attention, visual memory, and predictive decision - making ability). Twenty studies indicated the correlation between SVT outcomes and sport - specific or overall motor performance indicators (including hand - eye coordination, motor reaction ability, motor control ability, dynamic visual acuity, and multiple - object tracking ability), etc.

### 3.3 Effects of stroboscopic visual training on cognitive function

The reviewed studies demonstrated that SVT has a significant impact on various domains of cognitive function. A summary of the research protocols and main findings is provided in Table 1.

#### 3.3.1 Information processing speed

Among the three studies focusing on information processing speed, consistent improvements were observed. Football athletes showed enhanced ball control and goalkeepers exhibited faster information-processing speed after SVT (Elhachemi and Issam, 2024). Adolescent volleyball players in the stroboscopic group significantly improved in simple movement time, complex reaction speed, and reactive agility, with benefits largely maintained at a 4-week follow-up (Zwierko M. et al., 2023). A key finding was that the reduction in visual-motor reaction time observed in badminton athletes was primarily driven by a decrease in pre-movement time, rather than the movement execution time itself (Hülsdünker et al., 2021a).

#### 3.3.2 Visual attention

Analysis of four studies on visual attention revealed that SVT can significantly enhance players’ visual perception, attention allocation, and anticipation timing in sport-specific contexts (Ballester et al., 2017), such as ice hockey (Mitroff et al., 2013). However, its effects on peripheral vision and sustained attention were found to be limited (Appelbaum et al., 2011). Under stroboscopic conditions, a decline in passing accuracy was more pronounced among high-skilled athletes compared to their lower-skilled counterparts (Beavan et al., 2021). Conversely, other evidence indicated that skilled athletes can maintain performance by dynamically shifting their attention focus between external environmental and internal technical cues when visual feedback is restricted (Müller S, et al. (2024)).

#### 3.3.3 Visual memory

Emerging research supports the concept of visual memory plasticity, demonstrating that the capacity of visual memory can be modified through targeted training interventions. One study specifically assessed visual memory and found that the stroboscopic group demonstrated superior short-term memory retention under longer stimulus-cue intervals. Notably, this improvement persisted for at least 24 h, suggesting a degree of endurance in the training effects (Appelbaum et al., 2012).

#### 3.3.4 Predictive and decision - making abilities

A growing body of research has investigated the role of SVT in enhancing athletes’ predictive and decision-making abilities. Stroboscopic training was found to influence dribbling performance by reducing the number of ball touches, though this effect on dribbling precision was transient and rebounded after 1 week (Palmer et al., 2022). Participants exhibited reduced cognitive load during predictive tasks following SVT (Fortes et al., 2025). Significant improvements were also reported in perception-cognition test performance, sport-specific decision-making ability, and decision consistency (Top, 2022). In tennis, athletes who underwent low-frequency SVT (1 Hz) exhibited significantly higher accuracy in predicting serve directions (Boiangin, 2019). A single-session intervention on elite curling athletes adversely affected time perception and judgment, but a subsequent 4-week SVT intervention significantly enhanced the athletes’ time judgment and throwing speed control, though not throwing accuracy (Li et al., 2024a; 2024b).

### 3.4 Effects of stroboscopic visual training on motor performance

Evidence synthesized in this review confirms the beneficial role of SVT across various facets of motor performance, as detailed in Table 2.

#### 3.4.1 Effects of SVT on hand - eye coordination

Hand–eye coordination refers to the ability to direct hand movements based on visual input. In sports, it is primarily associated with ball-catching skills. SVT has been shown to improve performance in both ball-catching and ball-hitting, offering promising potential for skill transfer in training. Long-term stroboscopic visual control training significantly improved the shooting accuracy of elite female basketball athletes (Oudejans, 2012). Collegiate baseball batters in the experimental group exhibited significant improvements in launch angle and hitting distance compared to the placebo group, although these practice-based improvements did not extend to actual in-game performance (Liu et al., 2020). A single-session SVT intervention significantly improved participants’ hand-eye coordination, with enhancement evident immediately after training, as well as at 10-min and 10-day follow-up assessments (Ellison et al., 2020). Long-term stroboscopic training also improved the ability of softball players to field ground balls, with novice players exhibiting greater gains compared to expert athletes (Edgerton, 2018). Tests on tennis players showed that as the stroboscopic frequency decreased and the dark-phase ratio increased, target-hitting accuracy scores declined significantly (Jendrusch et al., 2023).

#### 3.4.2 Motor reaction ability

Motor reaction is a key indicator of motor performance. Wilkins et al. (2018) demonstrated that participants in the stroboscopic group exhibited consistent improvements in visual reaction time in both the post-test and the 4-week retention test, whereas no such improvements were observed in the control group. And long-term stroboscopic intervention significantly improved reaction agility and speed in young volleyball players (Zwierko M. et al., 2024). A single session of SVT increased reaction time across participants with different dribbling abilities, with the most substantial performance decline observed in high-ability players (Fransen et al., 2017). Conversely, a short-term stroboscopic intervention during the pre-game warm-up of elite athletes did not produce any immediate negative effects on visual-motor reaction performance (Strainchamps et al., 2023). The short-term application of stroboscopic glasses during soccer warm-up significantly improved performance in reactive agility tasks involving the ball, and this enhancement was maintained regardless of fatigue status (Zwierko T. et al., 2024).

#### 3.4.3 Postural control ability

SVT promotes rapid neural adjustments and responsiveness, thereby positively contributing to balance training (McCreary et al., 2024). Research indicated that postural stability under stroboscopic visual conditions is comparable to that observed with eyes closed (Kim et al., 2017; Kim et al., 2020). When the somatosensory system was disrupted, stroboscopic glasses significantly altered visual reliance during postural control (Lee et al., 2022). A single stroboscopic session under low-frequency visual conditions significantly influenced postural stability (Jang, 2019). Thirty minutes of balance training with stroboscopic glasses not only enhanced dynamic balance performance but also improved the retention of balance skills, with most gains maintained after 2 weeks (Symeonidou and Ferris, 2022). For older adults, SVT transformed postural sway towards a more closed-loop process and facilitated sensory reweighting, reducing visual dependency (Chen et al., 2025; Tsai et al., 2022).

#### 3.4.4 Dynamic visual acuity and multiple - Object tracking ability

The visual system maintains tracking of a moving object despite trajectory changes or obstructions by analyzing its spatiotemporal characteristics. SVT was found to immediately enhance dynamic visual acuity and improve ball-catching performance during training, although no long-term improvements were observed in follow-up tests conducted 2 weeks later (Holliday, 2013). In multiple-object tracking tasks, performance in the stroboscopic group remained stable regardless of frequency changes, whereas performance in the control group declined as frequency decreased. In multiple-object avoidance tasks, participants in the stroboscopic group demonstrated superior learning effects (Bennett et al., 2018).

## 4 Discussion

The primary objective of this review was to synthesize the effects of SVT on cognitive function and motor performance. The findings from 35 experimental studies collectively indicate that SVT is an effective intervention for enhancing a range of perceptual-cognitive and motor skills. The core mechanism underlying these improvements appears to be the neural adaptation induced by intermittent visual occlusion, which optimizes the efficiency of the visual and visuomotor systems.

### 4.1 Integration of key findings and theoretical implications

The consensus across the reviewed literature robustly supports the efficacy of SVT in enhancing fundamental cognitive processes. The pivotal finding that SVT reduces pre-movement time rather than movement execution time (Hülsdünker et al., 2021a) provides a critical mechanistic insight. This strongly suggests that SVT acts predominantly on the central nervous system to accelerate decision-making and motor planning, rather than merely improving the speed of muscular contraction. This central effect provides a parsimonious explanation for its broad transfer to various sports skills, as optimized central processing benefits multiple downstream actions.

Our results align with and extend the “Sports Vision Pyramid” model (Kirschen and Laby, 2011). SVT appears to target the foundational and middle layers of this pyramid—enhancing neural integration and processing of visual information—which in turn supports the attainment of optimal motor performance at the apex. The training challenges the brain to function more efficiently with less reliable visual input, thereby strengthening the neuro-visual foundations upon which sporting excellence is built.

However, the transfer of trained skills to authentic, competitive environments remains a critical consideration for practitioners. As evidenced by studies on baseball batting (Liu et al., 2020) and soccer dribbling (Palmer et al., 2022), improvements in controlled practice settings do not automatically translate to enhanced in-game performance. This underscores the notion that SVT should be viewed as a method to enhance foundational perceptual-cognitive capacities, which must then be strategically integrated into sport-specific, ecologically valid contexts to foster effective skill transfer.

### 4.2 Neuroplastic mechanisms and neural efficiency

The beneficial effects of SVT are not merely behavioral but are rooted in neuroplastic adaptations. The proposed mechanisms involve neural adaptations within the visuomotor system, including accelerated processing in motion-sensitive regions of the visual cortex (e.g., MT area) and enhanced neural efficiency (Hülsdünker et al., 2019; Wohl et al., 2024). Electrophysiological evidence, such as the reduction in P100 implicit time (Zwierko T. et al., 2023) and changes in N2 component latencies (Hülsdünker et al., 2021b; 2023), confirms that SVT accelerates early-stage visual processing and is associated with improvements in reaction speed.

The intermittent nature of visual input compels the brain to rely more heavily on visual-spatial memory and predictive extrapolation (Smith and Mitroff, 2012; Köster et al., 2023), thereby strengthening these higher-order cognitive pathways. Furthermore, findings of reduced blood-oxygen-level-dependent (BOLD) signal in key brain areas (e.g., left supramarginal gyrus, left inferior parietal lobule, left secondary somatosensory cortex, right superior frontal gyrus, and right supplementary motor area) after training (Wohl et al., 2024) provide support for the concept of neural efficiency—whereby the brain achieves superior performance with less effort or resource allocation after effective training.

### 4.3 Considerations for different populations and contexts

The effects of SVT are not uniform and are modulated by factors such as athletic proficiency and task demands. The observation that high-skilled athletes experience a more pronounced decline in passing accuracy under stroboscopic conditions (Beavan et al., 2021) suggests a greater dependency on continuous visual feedback to maintain precision at the elite level. However, their ability to maintain shooting accuracy by dynamically shifting attentional focus (Muller et al., 2024) also highlights their superior adaptive capacity.

The application of SVT extends beyond athletic enhancement into rehabilitation (Kroll et al., 2023). Promising evidence shows its utility in improving postural control in older adults by promoting sensory reweighting and reducing visual dependency (Chen et al., 2025; Tsai et al., 2022), and in aiding recovery for individuals with chronic ankle instability or ACL injuries (Avedesian, 2024; Mohess et al., 2024; Wohl et al., 2021). This underscores its potential as a versatile tool for both performance and therapy.

### 4.4 Limitations of the current body of evidence

Despite the promising evidence, this review highlights several significant limitations in the current literature. The most notable challenge is the lack of standardized training protocols. Substantial variability exists in stroboscopic frequencies, duty cycles, session durations, and intervention lengths across studies. This heterogeneity compromises the ability to compare results directly, establish dose-response relationships, and formulate evidence-based guidelines for practitioners.

Furthermore, the heavy reliance on behavioral outcomes provides limited insight into the underlying neural mechanisms. While a few studies have begun to incorporate neurophysiological measures, the field would greatly benefit from more extensive and multimodal investigations. Many studies are also confined to single-mode stroboscopic interventions, with insufficient exploration of how variables such as athletic level, stroboscopic frequency combinations, and integrated training approaches influence outcomes.

### 4.5 Implications for training practice

The findings of this systematic review provide clear guidance for the effective and safe application of SVT in athletic contexts. Based on the available evidence, we recommend that practitioners implement a long-term training regimen (≥4 weeks) utilizing a descending stroboscopic frequency paradigm, with a frequency of 2–3 sessions per week and 15 min of effective training time per session. The duty cycle should be appropriately set within the range of 50%–70%.

Training parameters should be individualized according to the specific demands of the sport and the athlete’s proficiency level. For elite athletes or precision-oriented sports (e.g., badminton), the use of relatively higher stroboscopic frequencies (e.g., 8–15 Hz) is recommended. In contrast, for dynamic, open-skill team sports (e.g., soccer) or athletes of moderate proficiency, lower frequencies (e.g., 1–6 Hz) are more suitable, particularly during the initial phases of training.

It is crucial to carefully manage the duty cycle and diligently monitor athletes’ fatigue levels throughout the implementation process. This practice is essential for maximizing training benefits and mitigating potential risks, such as dizziness or mental fatigue.

### 4.6 Future research directions

**FIGURE 5:**
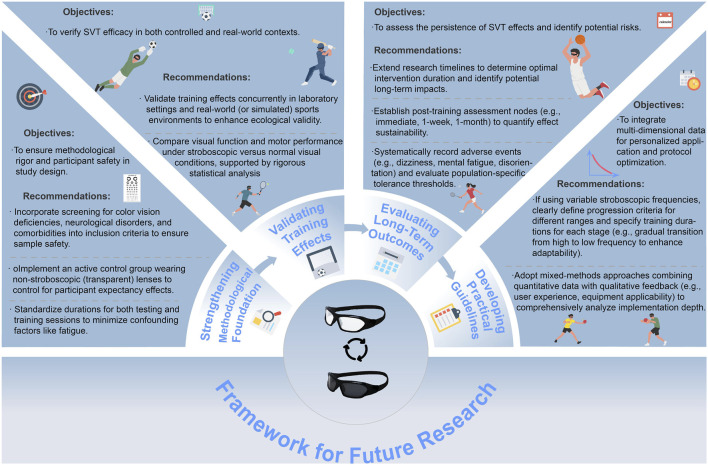
Proposed framework and recommendations for future research on Stroboscopic Visual Training (SVT). The figure summarizes a comprehensive set of methodological suggestions aimed at enhancing the rigor, ecological validity, safety, and practical application of SVT studies. Key recommendations include improving inclusion criteria, validating effects in real-world settings, implementing rigorous control procedures, standardizing protocols, and expanding research on long-term sustainability and potential adverse effects.

To address these limitations and unlock the full potential of SVT, we propose a comprehensive framework and set of recommendations aimed at enhancing the methodological rigor, ecological validity, and practical applicability of future SVT research, as illustrated in Figure 5.

Future studies may integrate portable devices to collect and transmit real-time physiological data—such as heart rate variability, reaction time, and muscle activation—during SVT interventions. By combining these data with individual training outcomes and leveraging big data analytics, researchers can establish a digital and intelligent dose–response model of SVT’s effects on cognitive function and motor performance. Additionally, advanced neuroimaging techniques, such as functional magnetic resonance imaging (fMRI), electroencephalography (EEG), and near-infrared spectroscopy (NIRS), should be employed to investigate, from a multimodal perspective, how SVT influences brain structure and function, and how these neural changes translate into behavioral improvements.

## 5 Conclusion

In conclusion, this systematic review establishes SVT not merely as a visual tool, but as a potent neurocognitive intervention. Its unique value lies in its “top-down” mechanism of action: by challenging the brain with intermittent visual input, SVT directly enhances foundational cognitive processes like information processing speed and attention allocation. These cognitive gains are not the ultimate goal but the critical mediator that drives the improvement of motor performance—from reactive agility to precise hand-eye coordination.

Therefore, the key practical implication of this study is the establishment of a new approach: “training the brain to optimize the body.” In practical application, it is essential to abandon a “one-size-fits-all” method and instead adopt scientifically-designed, individualized training protocols. The future of SVT lies in standardizing these protocols and further elucidating their underlying neural mechanisms, thereby developing it into an indispensable, scientifically-grounded method for enhancing sports performance.
